# The improving outcomes in intermittent exotropia study: outcomes at 2 years after diagnosis in an observational cohort

**DOI:** 10.1186/1471-2415-12-1

**Published:** 2012-01-18

**Authors:** Deborah Buck, Christine J Powell, Jugnoo Rahi, Phillippa Cumberland, Peter Tiffin, Robert Taylor, John Sloper, Helen Davis, Emma Dawson, Michael P Clarke

**Affiliations:** 1 Institute of Neuroscience, Newcastle University, Newcastle upon Tyne, UK; 2 Royal Victoria Infirmary Eye Dept, Newcastle upon Tyne Hospitals NHS Trust, UK; 3 Institute of Child Health, University College London, UK; 4 Sunderland Eye Infirmary, Sunderland, UK; 5 York Hospitals NHS Trust, York, UK; 6 Moorfields Eye Hospital, London, UK; 7 Academic Unit of Ophthalmology and Orthoptics, University of Sheffield, Sheffield, UK

## Abstract

**Background:**

The purpose of this study was to investigate current patterns of management and outcomes of intermittent distance exotropia [X(T)] in the UK.

**Methods:**

This was an observational cohort study which recruited 460 children aged < 12 years with previously untreated X(T). Eligible subjects were enrolled from 26 UK hospital ophthalmology clinics between May 2005 and December 2006. Over a 2-year period of follow-up, clinical data were prospectively recorded at standard intervals from enrolment. Data collected included angle, near stereoacuity, visual acuity, control of X(T) measured with the Newcastle Control Score (NCS), and treatment. The main outcome measures were change in clinical outcomes (angle, stereoacuity, visual acuity and NCS) in treated and untreated X(T), 2 years from enrolment (or, where applicable, 6 months after surgery). Change over time was tested using the chi-square test for categorical, Wilcoxon test for non-parametric and paired-samples t-test for parametric data.

**Results:**

At follow-up, data were available for 371 children (81% of the original cohort). Of these: 53% (195) had no treatment; 17% (63) had treatment for reduced visual acuity only (pure refractive error and amblyopia); 13% (50) had non surgical treatment for control (spectacle lenses, occlusion, prisms, exercises) and 17% (63) had surgery. Only 0.5% (2/371) children developed constant exotropia. The surgically treated group was the only group with clinically significant improvements in angle or NCS. However, 8% (5) of those treated surgically required second procedures for overcorrection within 6 months of the initial procedure and at 6-month follow-up 21% (13) were overcorrected.

**Conclusions:**

Many children in the UK with X(T) receive active monitoring only. Deterioration to constant exotropia, with or without treatment, is rare. Surgery appears effective in improving angle of X(T) and NCS, but rates of overcorrection are high.

## Background

Intermittent distance exotropia [X(T)] is a form of early onset childhood strabismus, affecting around 32 per 100,000 of children aged under 19 years [1]. It comprises periodic divergent misalignment [2,3] which is initially present on distance fixation, or during periods of tiredness or inattention alone, but may become more frequent and be present on near fixation, eventually leading to constant exotropia in some cases. Constant exotropia following de-compensation of intermittent to constant exotropia causes loss of near stereopsis, with suppression or panoramic vision and amblyopia, or diplopia, depending on age of onset.

The frequency with which X(T) deteriorates to constant exotropia is unclear, with some reports suggesting a high frequency and advocating early intervention [4,5], while others describe stability of X(T) over time in many patients [6-8].

Surgical treatment is performed to prevent deterioration to constant exotropia, to improve distance stereoacuity [9], and for aesthetic considerations. However it is known that surgical correction can result in overcorrection, causing constant esotropia, with potentially greater functional and clinical consequences than the original X(T) [10]. There is a lack of consensus on the appropriate timing of surgical treatment [11-15] which balances the risks of developing constant exotropia against those of persistent post-operative overcorrection.

In the absence of robust evidence to guide management it is difficult for clinicians to offer clear advice to the parents of children with X(T) [16,17]. The objective of this study was to investigate the current patterns of management and outcomes of X(T) in the UK.

## Methods

### Participants

Children under the age of 12 years, diagnosed with X(T) (of the true and simulated divergence excess and basic types) within the preceding 12 months and previously untreated, were eligible for the study. The minimum distance angle for inclusion was 10 prism diopters (PD). Children with convergence insufficiency type of intermittent exotropia (near deviation at least 10 PD more than distance deviation), constant exotropia, or significant coexisting ocular pathology such as cataract, were not eligible.

### Enrolment

Between May 2005 and December 2006 in 26 participating UK centres (see additional file 1: Collaborating centres), written informed consent was obtained from the parents/guardians of patients participating in this study. Details of the cohort and data collection procedures have been reported elsewhere [18].

### Procedure

No criteria were set regarding management decisions. Rather, treatment regimes were at the discretion of the local ophthalmologist/orthoptist, reflecting current practice in collaborating centres.

After piloting, a standardised assessment protocol [19] was followed in each centre which comprised ophthalmic examination at enrolment and orthoptic assessments at 3-monthly intervals within the first 12 months, and 6-monthly thereafter. A standardised clinical history was taken for all subjects, which included details of pregnancy, birth, general and ocular health, estimated age of onset, and family history of strabismus. Examination findings recorded included LogMAR visual acuity; total near and distance angle of strabismus using the alternate prism cover test and near stereoacuity using the Frisby Near Stereoacuity Test (FNS™). Control of the strabismus was assessed using the revised Newcastle Control Score (NCS) [20] which combines an estimate of observed frequency of the strabismus by parents/carers (home control) with an assessment of the child's ability to realign the eyes following a cover test to induce misalignment (clinic control), and is based on previously published recommendations for surgical treatment [4]. Possible NCS scores range from 0 to 9 (0 to 3 home control, 0 to 6 clinic control), with higher scores indicative of a worse control. While clinic control of X(T) may be unstable over short time periods [21], the parent reported element (home control) of the Newcastle Control Score does ensure that the parent's perspective is also taken into account: this is important in an era of patient-centred health care and outcomes and the pursuit of patient satisfaction.

Treatment was recorded as: observation only; treatment for reduced visual acuity only (pure refractive error and amblyopia); non-surgical treatment for control of X(T) (spectacle lenses, exercises, prisms, alternate day occlusion) and surgery for X(T).

### Measurement outcomes

All measurements were attempted for all participants within the constraints of age, cooperation and clinic time. Within this report near stereo data are reported only on participants aged 4 years or older because younger participants were unable to consistently complete testing. Distance stereoacuity testing was only possible in a limited number of centres, and is not reported.

For cases who received only observation or non-surgical interventions, the outcome measures reported here are those obtained at 24 months following enrolment (with a time window of ± 3 months). In cases where surgery was performed within 24 months following enrolment, the outcome measures reported here are those obtained at 6 months post-surgery (time window ± 3 months): this was considered the most suitable time point for assessment of outcomes within the time constraints of the study.

Measurements of the amount of change in angle and NCS excluded participants who had a persistent post-surgical overcorrection (NCS is not measurable in the presence of esotropia). Overcorrection was defined as the presence of a manifest esotropia (any amount) at 1/3 meter, 6 meters or both at 6 months post-surgery.

Constant exotropia was defined as a NCS clinic score of 6: 3 for near clinic control (constant exotropia at near), and 3 for distance clinic control (constant exotropia at distance), with absent stereoacuity where it was possible to test this. In children too young to perform stereoacuity testing, the absence of binocular functioning was determined by the absence of motor fusion as indicated by the use of prisms.

### Analysis and Statistical Methods

As treatments were not mutually exclusive, for the purposes of analysis participants who had treatment to both improve acuity and strabismus were classified as having had treatment for strabismus, and those participants who had non-surgical treatment prior to strabismus surgery were classified in the surgery group. Thus outcomes were compared by treatment group (observation, treatment for vision only, non-surgical treatment only for strabismus, surgical treatment for strabismus). Mean total NCS, home control and clinic control component scores were analysed. In terms of change in total NCS, we deemed a change of 3 or more to constitute deterioration or improvement in NCS control.

Change over time within groups was tested using the chi-square test for categorical, Wilcoxon test for non-parametric and paired-samples t-test for parametric data. Stereo data was transformed into log seconds of arc, with the few participants unable to respond to the highest level (400 seconds of arc) allocated a score at the next highest log level i.e. 2.90 log seconds of arc [22]. Change was then calculated on a linear scale for each individual and the change over time summarised for each group (median logsec and interquartile range (IQR)) and tested against the null hypothesis of no change.

Data were entered into a Microsoft Access database and analyzed using SPSS for Windows Version 11. The study was approved by the UK North West Multi-Centre Research Ethics Committee. Each collaborating centre obtained local approval from their relevant NHS Trust R&D (Research and Development) Department. The study was conducted in accordance with the tenets of the Declaration of Helsinki.

## Results

**Figure 1:**
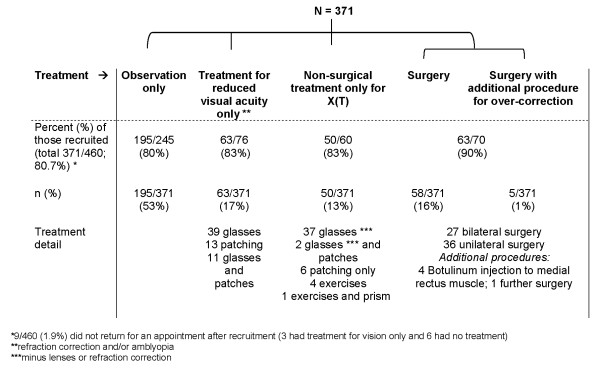
**Participation by treatment, received within 2 years of diagnosis**.

2 year follow-up data was available for 371/460 (81%) of participants, without significant differences in NCS and age at enrolment or treatment within 2 years, between those included and those lost to follow-up (Figure 1).

Of the 371 participants in the study, 195 (53%) had no treatment within two years, 63 (17%) had treatment for reduced visual acuity only, 50 (13%) had non-surgical treatment for X(T) whilst 63 (17%) participants had surgery within two years of enrolment. Figure 1 provides a breakdown of the types of treatment, and detail of participation by treatment received within 2 years. Only 2 children (1 male, 1 female) had constant exotropia at 2 year follow-up. These children are included in the main analysis. 13/63 (21%) of the surgical group had a persistent overcorrection at 6 months post-surgery.

### Newcastle Control Score (NCS)

The mean NCS at enrolment was higher (worse) in the surgical group (4.98) compared to the observation (3.43), vision only (3.38) and non-surgical treatment (4.02) groups (Table 1). At follow-up the total mean NCS in the surgery group was significantly reduced, on average more than 60% in both the parental and clinic component scores. There were significant but smaller reductions in the total mean NCS at follow-up in the non-surgical treatment and observation groups reflecting an average 20% reduction in the clinical component and 13% reduction in the parental component respectively.

**Table 1:** Change in mean and median total NCS by treatment group

	Treatment within 2 years:			
**Total NCS** **(0-9):**	**Observation** **(n = 191)**	**Vision only** **(n = 63)**	**Non-surgical treatment** **(n = 48)**	**Surgery** **(n = 49)**

Mean [SD] *median [IQR]* and (range) of NCS at enrolment	3.43 [1.44] *3 [2 to 4]* (1 to 8)	3.38 [1.53] *3 [2 to 4]* (1 to 7)	4.02 [1.47] *4 [3 to 5]* (1 to 9)	4.98 [1.94] *5 [3 to 6]* (2 to 9)
Mean [SD] *median [IQR] * and (range) of NCS at outcome	3.13 [1.65] *3 [2 to 4]* (0 to 9)	3.40 [1.89] *3 [2 to 4]* (0 to 7)	3.42 [2.01] *3 [2 to 4.75]* (0 to 8)	1.92 [2.19] *1 [0 to 3]* (0 to 9)
Mean difference[95% CIs]	-0.30[-0.55 to -0.05]	0.02[-0.41 to 0.44]	-0.60 [-1.15 to -0.06]	-3.06[-3.83 to -2.29]
Significance of difference between enrolment and 24 months/6-month post-op, by treatment group	p = 0.021* p = 0.028†	p = 0.94* p = 0.98†	p = 0.031* p = 0.026†	p < 0.001* p < 0.001†

* paired samples t-test for differences in means

† Wilcoxon test for differences in medians

Changes in NCS from enrolment to follow-up are shown by treatment group in Table 2. Overall in the observation group, 19 (10%) improved, 163 (85%) stayed stable and 9 (5%) deteriorated; in the non-surgical treatment group 7 (15%) improved, 39 (81%) stayed stable and 2 (4%) deteriorated; in the treatment for vision only group, 5 (8%) improved, 53 (84%) stayed stable and 5 (8%) deteriorated; in the surgery group 26/62 (42%) improved, 21 (34%) stayed stable, 2 (3%) deteriorated while 13 (21%) were overcorrected.

**Table 2:** Change in total Newcastle Control Score by treatment group

	Treatment within 2 years:			
	Observation n = 191	Vision only n = 63	Non-surgical treatment n = 48	Surgery n = 62
Improved by ≥3; n (%)	19 (10)	5 (8)	7 (15)	26 (42)
Stable; n (%)	163 (85)	53 (84)	39 (81)	21 (34)
Deteriorated by ≥3; n (%)	9 (5)	5 (8)	2 (4)	2 (3)
*Overcorrected n (%)*	*-*	*-*	*-*	*13 (21)*

The home control component score was missing for 1 patient at enrolment and outcome NCS were missing for one surgery and 5 non-surgery patients because the home control component was not completed. (For the child who had surgery, no detectable squint was seen in the clinic.) Difference between groups in proportions improved/stable/deteriorated: chi-square 58.9, p < 0.001.

A score at final outcome of 0 (no detectable strabismus) was found in 15/62 (24%) of the surgery group plus a further 4/62 (6%) who received Botulinum toxin to treat overcorrection within 6 months of surgery, compared with 5/191 (2.5%) in the observation group, 1/48 (2%) in the non-surgical treatment group and 4/63 (6%) in the group treated only for reduced visual acuity.

There was no statistically significant difference in overcorrection rates by type of surgery (7/27 (26%) in the bilateral compared to 6/36 (17%) in the unilateral group; Chi-Square test 0.80, p = 0.37). Of the 13 overcorrections, mean [SD] total NCS, home control and clinic control at enrolment were 5.08 [1.71], 2.08 [0.86] and 3.0 [1.29] respectively (ranges 2 to 8, 1 to 3 and 1 to 6 respectively).

### Changes in angle of X(T)

Table 3 provides details of near and distance angle. At enrolment the median angle was 14 diopters for near (range -6 to 50) and 25 diopters for distance (range 10 to 50) in the non-surgical groups and did not vary over time. In the surgery group the median angle at enrolment was 16 for near (range 1 to 45) and 30 for distance (range 20 to 60), and there were clinically significant changes at follow-up: near angle was reduced from 16 to 7, and distance angle from 30 to 10 (Table 3).

**Table 3:** Change in near and distance angle by treatment group

	Treatment within 2 years:			
	Observation	Vision only	Non-surgical treatment	Surgery
**Near APCT**	n = 171	n = 59	n = 46	n = 40^†^
Median [IQR] at enrolment	14[10 to 20]	14[10 to 20]	14[10 to 18]	16 [10 to 24]
Median [IQR] at outcome	14 [10 to 20]	14[8 to 20]	14 [10 to 20]	7 [2 to 12]
Median change [IQR]	0[-4 to 4]	0[-4 to 5]	0[-2 to 4]	-9*[-18 to -1]

**Distance APCT**	n = 169	n = 58	n = 46	n = 42^†^
Median [IQR] at enrolment	25 [20 to 35]	25[20 to 30]	25[20 to 32]	30[25 to 35]
Median [IQR] at outcome	25[19 to 30]	25[18 to 32]	25[18 to 30]	10[5 to 18]
Median change [IQR]	0[-7 to 5]	0[-6 to 5]	0[-5 to 0]	-20*[-30 to -7]

^†^excludes overcorrections * p < 0.001 (Wilcoxon test)

### Near Stereoacuity

212 participants were aged 4 years or older at enrolment, of whom 166 (78%) attended follow-up. Log transformed measures of near stereoacuity were available for 150/166 (90%) at both enrolment and outcome (Table 4). There were significant improvements in the observation and non-surgical treatment groups. The surgery group overall also showed a similar level of improvement although this was not statistically significant. Of the 6 overcorrected children aged 4 or older at enrolment, 5 (1 with prism) had some stereoacuity at follow-up (with an abnormal head posture), and while stereoacuity was absent in the other, his/her stereoacuity at enrolment was unknown.

**Table 4:** Change in near stereoacuity (log transformed) by treatment group

	Treatment within 2 years:			
	Observation (n = 80)	Vision only (n = 25)	Non-surgical treatment (n = 25)	Surgery (n = 20)†
Median [IQR] logsec at enrolment	1.93 [1.74 to 2.04]	1.74 [1.74 to 2.08]	1.93[1.93 to 2.20]	1.93[1.74 to 2.14]
Median [IQR] logsec at outcome	1.74 [1.60 to 1.93]	1.74 [1.74 to 1.93]	1.74[1.60 to 1.90]	1.74[1.74 to 1.93]
Median change [IQR] in logsec	-0.23 [-0.45 to 0.0]	0[-0.35 to 0.0]	-0.33[-0.51 to 0.0]	-0.09[-0.50 to 0.0]
Significance of difference between enrolment and 24 months/6-month post-op by treatment group*	p < 0.001	p = 0.030	p < 0.001	p = 0.090

† Includes overcorrections * Wilcoxon test

### Visual Acuity

63/371 (17%) participants had treatment directed at improving visual acuity only (i.e. refractive correction and/or amblyopia treatment) within 2 years from enrolment, including 26 participants who were observed at enrolment and subsequently began treatment within 2 years of follow-up.

288/371 participants (78%) had LogMAR measures of visual acuity in each eye at both enrolment and outcome. There were small but statistically significant improvements in mean acuity in the worse eye for all groups apart from the surgical group, presumably reflecting maturational changes in the observation group (Table 5).

**Table 5:** Change in visual acuity (worse eye) between enrolment and outcome, by treatment group

	Treatment group:			
	Observation (n = 156)	Vision only (n = 45)	Non-surgical treatment (n = 40)	Surgery (n = 36)
Mean [SD] acuity(worse eye) at enrolment (LogMAR)	0.127 [0.104]	0.211 [0.117]	0.169 [0.142]	0.178 [0.145]
Mean [SD] acuity(worse eye) at outcome (LogMAR)	0.081 [0.083]	0.141 [0.127]	0.107 [0.134]	0.144 [0.133]
Mean change[95% CIs]	-0.045[-0.061 to-0.029]	-0.069[-0.105 to -0.035]	-0.062[-0.109 to -0.015]	-0.034[-0.085 to 0.018]
Significance of difference between enrolment and 24 months/6-month post-op by treatment group*	p < 0.001	p < 0.001	p = 0.011	p = 0.195

*Paired samples t-test

## Discussion

From a multicentre study we report that only 17% of a representative cohort of 371 children with X(T) were treated surgically within 2 years of presentation. More than half (53%) of the cohort were observed without any treatment and 17% were treated for reduced acuity rather than for strabismus. The proportion of children who were simply observed is higher than would be anticipated from some of the prior literature that advocates early surgical treatment [4.5,11-13] possibly reflecting international variations in practice. Our findings suggest that observation without intervention is not associated with deterioration in clinical outcomes in the majority of children, in particular there appears to be an extremely low conversion rate to constant exotropia (0.5%) within the first 2 years after diagnosis. While this finding is supported by studies which have found X(T) to be a relatively stable condition in many cases [6-8] it is possible that more children in our study may have developed constant exotropia had they not received treatment.

The children who underwent surgery had worse control at presentation, as measured by the Newcastle Control Score (which was expected as this is based on criteria for surgical treatment). However they did not have significantly greater angles of strabismus than those who were treated non-surgically or simply observed. While we cannot rule out potential bias in comparing outcome at 6 months post-operatively with 24 month from enrolment in those not operated on, surgery appeared the most effective intervention in reducing the angle of X(T) and improving scores on the NCS, albeit with a risk of overcorrection and, in one case, absence of near stereoacuity.

Although prospective and involving a large number of centres and with a reasonable sample size, (thus minimising selection bias and role of chance), the observational nature of our study does not offer the same possibility for comparing the effectiveness of different treatment strategies as a randomised controlled trial. Nevertheless, with standardised data collection and a good level of completeness of follow-up, our study has strengths in relation to understanding current management practices in the UK and their effect on outcomes.

One reason for the low rates of surgery in our study could have been concern about the possibility of persistent post-operative overcorrection. 13/63 (21%) of the patients undergoing surgery had persistent overcorrections at 6 months following surgery, including one who had had a medial rectus injection of Botulinum toxin between surgery and 6 months follow-up. A further 4 patients had persistent overcorrections treated successfully with either Botulinum toxin or surgery prior to the 6 month follow-up period. Ekdawi et al [23] reported that 12 of 61 (19.7%) of their sample underwent a second surgery, however in only 2 cases was this for consecutive esotropia, the remainder of the second surgeries being for recurrent exotropia. Likely explanations for the high overcorrection rate in the present study include the relatively short follow-up period, the more stringent classification of an overcorrection and possible variation in practice between individual surgeons.

Non-surgical treatment of X(T) had less significant impact on angle of deviation or scores on the NCS, in keeping with some prior reports [24,25], although greater impact has been reported by others [26]. Whilst no clinically significant changes were noted in angle of deviation or on the NCS in children who were simply observed, small statistically significant changes in near stereoacuity and visual acuity were seen in this group and serve to underline the importance of normal developmental changes in visual function that occur with age which need to be accounted for in studies of natural history and outcomes. We were unable to report distance stereoacuity and this is an important limitation of the study.

The proportion of children receiving treatment for reduced visual acuity (rather than strabismus) was surprisingly large and almost all of these had refractive correction. A minority received patching in addition; most children treated in this way had minor reductions in acuity. Overall, no significant effects were observed in median angle of X(T) or in NCS from treatment directed at improving visual acuity alone.

## Conclusions

The findings of the present study highlight that: a) many children with X(T) do not experience adverse outcomes from observation or non-surgical treatment; b) the risk of conversion from intermittent to constant exotropia is minimal; c) a significant proportion of children who undergo surgery for X(T) experience an overcorrection, occasionally with loss of near stereoacuity.

## Abbreviations

IQR: is used to abbreviate 'interquartile range'; NCS: is used for 'Newcastle Control Score'; PD: is used for 'prism diopter'; and X(T): is used to abbreviate 'intermittent distance exotropia'.

## Competing interests

The authors declare that they have no competing interests.

## Authors' contributions

DB contributed to study design and coordination, conducted the data analysis, and assisted in drafting and revising the manuscript. CJP was involved in data acquisition, data interpretation, and critical revision of the manuscript. JR helped to interpret the data and to draft and revise the manuscript. PC provided statistical advice and helped to interpret the data and to draft/revise the manuscript. PT, RT and JS assisted with data acquisition and interpretation, and critical revision of the manuscript. HD contributed to study design, data interpretation and manuscript revision. ED was involved in data acquisition and revision of the manuscript. MPC conceived the study, participated in its design, and helped to interpret the data and draft/revise the manuscript. All authors have given final approval of the version to be published.

## Pre-publication history

The pre-publication history for this paper can be accessed here:

http://www.biomedcentral.com/1471-2415/12/1/prepub

## Supplementary Material

Additional file 1 **The IOXT study collaborating centres**. a list of the 26 collaborating centres. Click here for file
