# Supplementary material for: The improving outcomes in intermittent exotropia study: outcomes at 2 years after diagnosis in an observational cohort
Source: BMC Ophthalmol. 2012 Jan 18;12:1. doi: 10.1186/1471-2415-12-1 (PMC3293086; doi:10.1186/1471-2415-12-1)
Supplement: Additional file 1 — The IOXT study collaborating centres. a list of the 26 collaborating centres. [file 1471-2415-12-1-S1.DOC]

**The IOXT study collaborating centres**

| Birmingham Children’s Hospital, Eye Dept, **Birmingham, UK** |
| --- |
| Bradford Royal Infirmary, Bradford, UK |
| Sussex Eye Hospital, **Brighton, UK** |
| Bristol Eye Hospital, **Bristol, UK** |
| Queen’s Hospital, Orthoptics Dept, **Burton upon Trent**, UK |
| West Suffolk Hospital, **Bury St Edmunds, UK** |
| Ninewells Hospital, **Dundee, UK** |
| University Hospital of North Durham, Orthoptic Dept, Durham, UK |
| The Princess Alexandra Eye Pavilion, Edinburgh, UK |
| Royal Devon & Exeter Hospital, West of England Eye Unit, Exeter, UK |
| Hull & East Yorkshire Eye Hospital, Kingston upon Hull  and  Hull Royal Infirmary, Orthoptic Dept, Hull, UK |
| St James's University Hospital, Orthoptic Dept, Leeds, UK |
| Moorfields Eye Hospital, London, UK |
| North Middlesex University Hospital, Orthoptic Dept, London, UK |
| Manchester Royal Eye Hospital, Orthoptic Dept, Manchester, UK |
| Milton Keynes General Hospital, Milton Keynes, UK |
| Royal Victoria Infirmary, Eye Dept, Newcastle upon Tyne, UK |
| Royal Shrewsbury Hospital, Orthoptics Dept, Shrewsbury  and  Princess Royal Hospital, Telford, UK |
| Southampton General Hospital, Eye Unit, Southampton, UK |
| Sunderland Eye Infirmary, Sunderland, UK |
| Mayday University Hospital, Eye Unit, Thornton Heath, UK |
| Torbay District General Hospital, Orthoptics Dept, Torquay, UK |
| Christopher Home Eye Unit, Orthoptics Dept, Royal Albert Edward Infirmary, Wigan, UK |
| Singleton Hospital, Orthoptic Dept, Swansea, UK |
| University Hospital of Wales, Cardiff, UK |
| York Hospital, Ophthalmology Dept, York, UK |
